# Supplementary material for: DDX3Y is likely the key spermatogenic factor in the AZFa region that contributes to human non-obstructive azoospermia
Source: Commun Biol. 2023 Mar 31;6:350. doi: 10.1038/s42003-023-04714-4 (PMC10063662; doi:10.1038/s42003-023-04714-4)
Supplement: Supplementary file 2 — Supplementary Information [file 42003_2023_4714_MOESM2_ESM.pdf]

Supplementary Information

Table of Contents

Supplementary Table 1. ACMG-AMP classification of *DDX3Y* variants.

Supplementary Table 2. Primer information.

Supplementary Data 1. Gene disease curation.

Supplementary Figure 1. Calculation of relatedness between M2171 and male relatives.

Supplementary Figure 2. Functional characterization of *DDX3Y* c.1609+1del by Minigene assay.

Supplementary Figure 3. Sanger traces of c.1230_1231del (ESTNAND cohort patient GEMINI-492).

Supplementary References

Supplementary Table 1: ACMG-AMP classification of *DDX3Y* variants.

Patient ID	Gene	Variant c.	Variant p.	Genotype	MAF (gnomAD)	Classification	Applicable ACMG criteria	Associated phenotype
M3086	*DDX3Y*	c.428dup	p.(Glu145Glyfs*13)	hem.	-	Class 4	PVS1_S, PM2, PP4	SCO
GEMINI-492	*DDX3Y*	c.1230_1231del	p.(Asn412Hisfs*13)	hem.	-	Class 4	PVS1_S, PM2	NOA
M2185	*DDX3Y*	c.1272dup	p.(Lys425*)	hem.	-	Class 4	PVS1_S, PM2, PP4	SCO
M2171	*DDX3Y*	c.1609+1del	p.(Gly537Alafs*12)	hem.	-	Class 4	PVS1_S, PS2_M, PM2, PP4	SCO

Supplementary Table 2: Primer information.

***DDX3Y* Primer**	
**Sequencing primer**	
c.428dup	
Forward	5’-TGAAAGACCTGGCTTTGGCA-3’
Reverse	5’-AGGACAGTTACTGCCGGTTG-3’
c.1230_1231del	
Forward	5'-TGAACAAGATACTATGCCACCA-3'
Reverse	5'-GGCTTAATTCTAGTCCTTGTGC-3'
c.1272dup	
Forward	5'-TCAGATGCTTGCTCGTGACT-3'
Reverse	5'-GGATGCATGCAAACAGAGCC-3'
c.1609+1del	
Forward	5'-GCAGCACGAGGACTAGACAT-3'
Reverse	5'-GGCAAGGCCTAAAGTGTTCA-3'
**Primer used in minigene assay**	
c.1609+1del (Initial amplification)	
Forward	5'-CACCCTTCACCAGTTTCGCTCAGG-3'
Reverse	5'-AAAGAATGAGGTGGCAAGGC-3'
Minigene exon primer (Rat *INS2* exons 3 and 4)	
Forward	5'-CCTGCTCATCCTCTGGGAGC-3'
Reverse	5'-AGGTCTGAAGGTCACGGGCC-3'

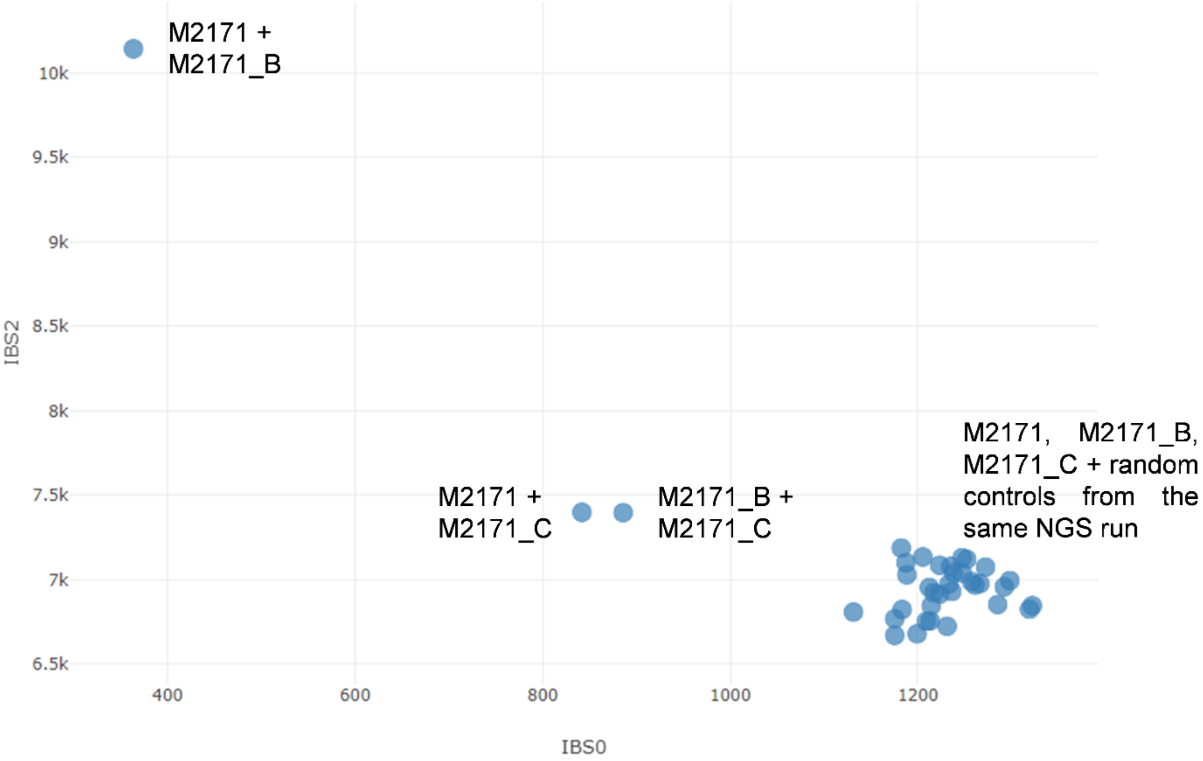

Supplementary Figure 1. Calculation of relatedness between M2171 and male relatives. Relatedness of M2171 and his brother M2171_B and cousin M2171_C was calculated by exome sequencing and subsequent analysis using the Somalier tool[1]. One point represents the relatedness of two samples (as indicated). IBS0 (x-axis) is the number of positions, where one sample is homozygous for the reference and another is homozygous for an alternate nucleotide. IBS2 (y-axis) is the number of sites where samples have the same genotype. Therefore, unrelated control samples cluster on the bottom right, whereas the patient and his brother are highly related, which is indicated as a dot on the top left. M2171 and his cousin locate in between, showing a closer relation than random samples but not as close as brothers.

a)

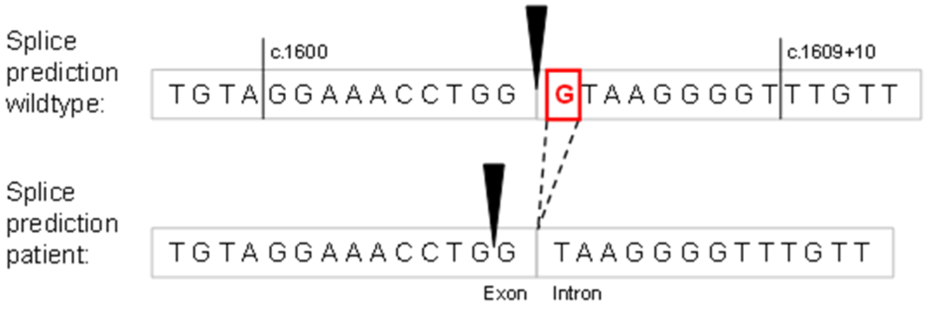
Splice prediction wildtype:
c.1600
c.1609+10
TGTA GGAAACCTGG G TAAGGGGT TTGTT
Splice prediction patient:
TGTAGGAAACCTGG TAAGGGGTTTGTT
Exon
Intron

b)

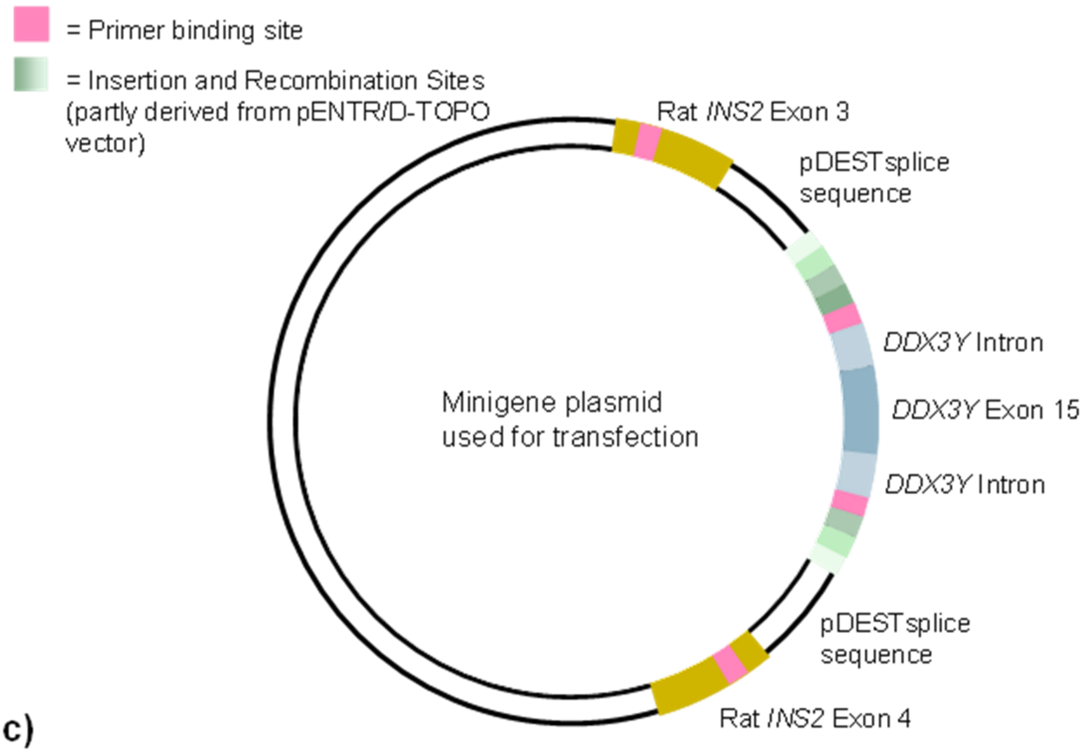
= Primer binding site
= Insertion and Recombination Sites (partly derived from pENTR/D-TOPO vector)
Rat *INS2* Exon 3
pDEST splice sequence
DDX3Y Intron
DDX3Y Exon 15
DDX3Y Intron
pDEST splice sequence
Rat *INS2* Exon 4
Minigene plasmid used for transfection

c)

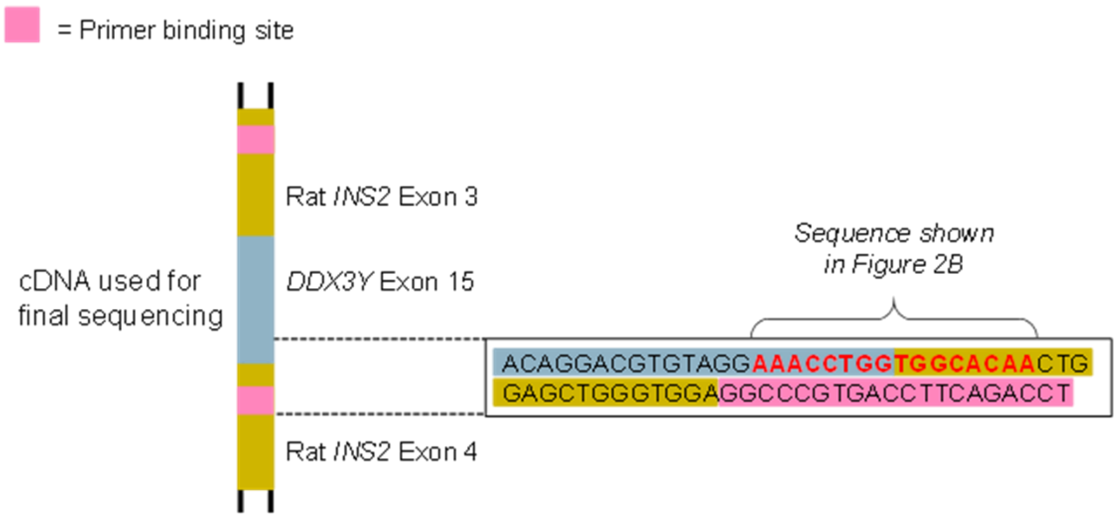
= Primer binding site
Rat *INS2* Exon 3
DDX3Y Exon 15
Rat *INS2* Exon 4
cDNA used for final sequencing
Sequence shown in Figure 2B
ACAGGACGTGTAGGAAACCTGGTGGCACAACTG
GAGCTGGGTGGAGGCCCGTGACCTTCAGACCT

Supplementary Figure 2. Functional characterization of *DDX3Y* c.1609+1del by minigene assay.

a) The splice site prediction tools Splice Site Finder (SSF-like)[2], MaxEntScan (MES)[3] and Splice Site Prediction by Neural Network (NNPLICE)[4] accessed through Alamut Visual Plus 1.4 (Interactive Biosoftware) consistently predict a shifting of the splice site at c.1609+1 to c.1609. **b)** Expression vector containing the minigene construct was generated through initial amplification of exon 15 of *DDX3Y* and adjutant intronic sequences from the patient's as well as from a control's genomic DNA, subsequent cloning of the fragment into pENTR™/D-TOPO® and subcloning in pDESTsplice[5]. The expression construct contains a minigene consisting of exon 3 and 4 of the rat *Ins2* gene, intronic sequences and exon 15 of *DDX3Y*, and was used for transient transfection of HEK329 cells. **c)** Schematic depiction of the cDNA derived from total RNA of HEK cells 24 hours after transfection. Introns and cloning sites of the vector were spliced out, mimicking the *in vivo* splicing event in the patient. General primers binding within the exons of the rat *Ins2* gene were used to sequence the resulting cDNA.

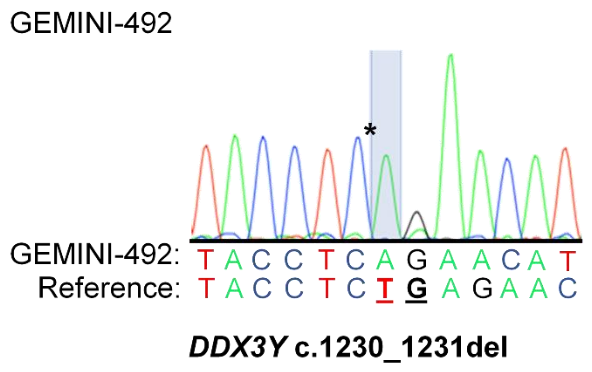

Supplementary Figure 3. Sanger traces of c.1230_1231del (ESTNAND cohort patient GEMINI-492).

Sequence traces of the recruited Estonian ESTNAND cohort patient (case GEMINI-492) showing the hemizygous variant *DDX3Y* c.1230_1231del. DNA sequence of the reference is [-TACCTC-**TG-**AGAACAT-] and of the frameshift variant detected in the patient is [-TACCTCAGAACAT-].

Supplementary References

1. Pedersen, B. S. *et al.* Somalier: Rapid relatedness estimation for cancer and germline studies using efficient genome sketches. *Genome Med.* **12**, 1–9 (2020).

2. Shapiro, M. B. & Senapathy, P. RNA splice junctions of different classes of eukaryotes: sequence statistics and functional implications in gene expression. *Nucleic Acids Res.* **15**, 7155–7174 (1987).

3. Yeo, G. & Burge, C. B. Maximum entropy modeling of short sequence motifs with applications to RNA splicing signals. *J. Comput. Biol.* **11**, 377–94 (2004).

4. Reese, M. G., Eeckman, F. H., Kulp, D. & Haussler, D. Improved splice site detection in Genie. *J. Comput. Biol.* **4**, 311–23 (1997).

5. Kishore, S., Khanna, A. & Stamm, S. Rapid generation of splicing reporters with pSpliceExpress. *Gene* **427**, 104–110 (2008).
