# Supplementary material for: DDX3Y is likely the key spermatogenic factor in the AZFa region that contributes to human non-obstructive azoospermia
Source: Commun Biol. 2023 Mar 31;6:350. doi: 10.1038/s42003-023-04714-4 (PMC10063662; doi:10.1038/s42003-023-04714-4)
Supplement: Supplementary file 3 — Description of Additional Supplementary Files [file 42003_2023_4714_MOESM3_ESM.pdf]

Description of Additional Supplementary Files

File name: Supplementary Data 1

Description: Gene Disease Curation
